# MORBIDITY AND SURVIVAL AFTER PERIOPERATIVE CHEMOTHERAPY IN GASTRIC CANCER: A STUDY USING THE BECKER’S CLASSIFICATION AND REGRESSION

**DOI:** 10.1590/0102-672020220002e1704

**Published:** 2023-01-09

**Authors:** Maria Cecília de Aguiar MACHADO, José Pedro Coimbra de Vargas Lobarinhas BARBOSA, Filipa Ferreira de OLIVEIRA, José Adelino Lobarinhas BARBOSA

**Affiliations:** 1 Universidade do Porto, Faculty of Medicine – Porto, Portugal;; 2 São João University Hospital, Department of Stomatology – Porto, Portugal;; 3 Universidade do Porto, Faculty of Medicine, Department of Surgery and Physiology – Porto, Portugal;; 4 São João University Hospital, Department of General Surgery – Porto, Portugal.

**Keywords:** Stomach Neoplasms, Drug Therapy, Lymph Nodes, Postoperative Complications, Survival, Neoplasias Gástricas, Tratamento Farmacológico, Linfonodos, Complicações Pós-operatórias, Sobrevida

## Abstract

**BACKGROUND:**

Gastric cancer is an aggressive neoplasm with a poor prognosis. The multimodal approach with perioperative chemotherapy is currently the recommended treatment for patients with locally advanced gastric cancer. This treatment induces a histopathological response expressed either through the degree of regression of the primary tumor or of the lymph nodes or through yTNM staging. Despite its advantages, there are still doubts regarding the effects of chemotherapy on postoperative morbidity and mortality.

**AIMS:**

This study aims to evaluate the impact of perioperative chemotherapy and its effect on anatomopathological results and postoperative morbidity and on patient survival.

**METHODS:**

This is an observational retrospective study on 134 patients with advanced gastric cancer who underwent perioperative chemotherapy and curative radical surgery. The degree of histological regression of the primary tumor was evaluated according to Becker’s criteria; the proportion of regressed lymph nodes was determined, and postoperative complications were evaluated according to the Clavien-Dindo classification. Survival times were compared between the groups using Kaplan-Meier curves and the Mantel-Cox log-rank test.

**RESULTS:**

In all, 22.3% of the patients were classified as good responders and 75.9% as poor responders. This variable was not correlated with operative morbidity (p=1.68); 64.2% of patients had invaded lymph nodes and 46.3% had regressed lymph nodes; and 49.4% had no lymphatic invasion and 61.9% had no signs of venous invasion. Postoperative complications occurred in 30.6% of the patients. The group of good responders had an average survival of 56.0 months and the group of poor responders had 34.0 months (p=0.17).

**CONCLUSION:**

Perioperative chemotherapy induces regression in both the primary tumor and lymph nodes. The results of the operative morbidity were similar to those described in the literature. However, although the group of good responders showed better survival, this value was not significant. Therefore, further studies are needed to evaluate the importance of the degree of lymph node regression and its impact on the survival of these patients.

## RESUMO

**RACIONAL:**

O cancer gástrico é uma neoplasia com mau prognóstico. A abordagem multimodal com quimioterapia-perioperatória é o tratamento recomendado para os pacientes com cancer gástrico localmente avançando. Este tratamento induz uma resposta histopatológica manifestado pelo grau de regressão do tumor primário, dos gânglios linfáticos e do estadiamento ypTNM. Apesar de suas vantagens, ainda há dúvidas quanto aos efeitos da quimioterapia na morbimortalidade pós-operatória.

**OBJETIVOS:**

Avaliar o impacto da quimioterapia perioperatória e o seu efeito nos resultados anatomopatológicos, na morbidade pós-operatória e na sobrevida.

**MÉTODOS:**

Estudo observacional-retrospetivo com 134 doentes com cancer gástrico avançado, que se submeteram a quimioterapia perioperatória e cirurgia radical curativa. O grau de regressão histológico do tumor primário foi avaliado de acordo com os critérios de Becker. A proporção de gânglios regredidos foi determinada e as complicações pós-operatórias foram avaliadas com a classificação de Clavien-Dindo. Os tempos de sobrevida foram comparados entre os grupos por meio das curvas de Kaplan-Meier e do teste Mantel-Cox Log Rank.

**RESULTADOS:**

22,3% dos doentes foram classificados como bons-respondedores e 75,9% como maus-respondedores. Esta variável e a morbidade pós-operatória não estavam relacionadas. 64,2% dos doentes apresentaram invasão ganglionar e 46,3% tinham regressão ganglionar, 49,4% não tinham invasão linfática e 61,9% não tinham sinais de invasão venosa. As complicações pós-operatórias ocorreram em 30,6% dos pacientes. O grupo dos bons respondedores apresentou uma sobrevida mediana de 56,0 meses e o grupo dos maus respondedores 34,0 meses.

**CONCLUSÕES:**

A quimioterapia perioperatória induz a regressão quer do tumor primário e dos gânglios-linfáticos. Os resultados da morbidade pós-operatória foram semelhantes aos descritos na literatura. Apesar do grupo dos bons-respondedores apresentar melhor sobrevida, este valor não foi significativo. Assim, são necessários mais estudos que avaliem a importância do grau de regressão ganglionar e o seu impacto na sobrevida.

## INTRODUCTION

Gastric cancer (GC) remains one of the biggest challenges in oncology, being the fifth most diagnosed neoplasm and the third most common cause of cancer-related deaths worldwide^1,11,17,19,26,27^. In Western countries, this high mortality is mostly related to the advanced stage of cancer at the time of diagnosis^1,4,6,17^.

There are several multimodal approaches for the treatment of GC, but in Western countries, the recommended approach for patients with locally advanced stage is perioperative chemotherapy (POC) together with radical surgery with curative intent^17^. The importance of POC was highlighted by the MAGIC and FNLCC/FFCD clinical trials, which showed an improvement in the survival rates of these patients when compared to those submitted to surgery alone^6^. More recently, the FLOT4 study recommended the use of POC as the gold standard for locally advanced GC^3^. It is acknowledged that POC prolongs the survival of these patients through the downstaging of both the primary tumor and lymph nodes (LNs), eliminates potential micrometastases, increases the rate of complete surgical resection, and provides information regarding the tumor’s chemosensitivity^10,11,16,17,21,23,26^.

On the contrary, studies report contradictory results regarding the effects of POC on operative morbidity and mortality. In fact, according to some authors, POC predisposes to an increased risk of postoperative complications^13^ and may also translate into a lower quality of life^23^. However, according to others, POC was not significantly associated with an increase in postoperative complications^23,26,27^, nor did it affect the long-term survival of patients^26^. In 2018, Claassen et al. published the CRITICS trial, in which they related an increase in operative morbidity^7^ *.*

POC induces a histopathological response that may be expressed through the tumor regression grading (TRG)^8,10,12,22^ and the assessment of the effects of TNM downstaging^10,20^. This response provides information regarding the tumor’s chemosensitivity and helps to predict prognosis^22^. The TRG is a better tool to evaluate the effects of POC since the TNM downstaging results from the comparison between clinical and pathological staging and, more recently, also post-neoadjuvant therapy staging — the yTNM^8,22^. Clinical staging is determined through the combination of endoscopic ultrasound and CT or MRI, which provide limited information regarding the T and N, often with little precision^10^. More recent studies have highlighted the importance of LN regression regarding the reduction in the death risk in locally advanced GC, in relation to the ypTNM^8,11,16^.

The objective of this study was to assess the impact of POC and its effect on anatomopathological results and operative morbidity. This study also aimed to evaluate the effect of POC on patient survival, according to the TRG.

## METHODS

This is a retrospective observational study in a casuistic of 134 patients with GC (including type III Siewert esophagogastric junction adenocarcinoma^15^), who underwent POC in the São João University Hospital (CHUSJ) from January 2011 to May 2020. Patients were identified through an examination of clinical records in CHUSJ’s electronic database. Eligibility criteria were as follows: histopathological evidence of gastric adenocarcinoma; locally advanced GC (8th edition AJCC cancer staging — cT2N1M0-T4N3M0, II–III); patients who underwent POC together with curative radical surgery; and age over 18 years. Exclusion criteria included patients not submitted to resection surgery.

### Diagnosis and treatment

Diagnosis and clinical staging were determined according to the results of endoscopy with biopsy, thoracoabdominopelvic CT, and diagnostic laparoscopy. The chemotherapy regimen and the number of cycles were determined by the multidisciplinary cancer group. Three weeks after the last cycle of chemotherapy, patients were submitted to subtotal or total gastrectomy with D2 lymphadenectomy. Patient follow-up was carried out according to ESMO’s recommendations^18^.

### Histopathological evaluation

The resected specimens were evaluated by the pathology department. The TNM stage was determined according to the TNM classification of the AJCC, 8th edition. The TRG was determined according to Becker’s criteria,^5^ which are based on the percentage of residual microscopic tumor in relation to the macroscopically identified tumor. It is classified into three grades: TRG 1a – indicates complete tumor regression (absence of tumor) and TRG 1b – subtotal tumor regression (<10% of residual tumor); TRG 2 – partial tumor regression (10–50% of residual tumor); and TRG 3 – minimal or absent tumor regression (>50% of residual tumor). The proportion of regressed LNs was calculated through the formula:

$$\frac{\text{Regressed lymph nodes}}{\text{Invaded lymph nodes + Regressed lymph nodes}}$$

Postoperative complications were evaluated according to the Clavien-Dindo classification.

### Statistical analysis

Data were analyzed using IBM SPSS^®^ Statistics, version 27. Continuous variables were assessed for normality by visual analysis of their histograms and described using their median and interquartile range. Categorical variables were described with absolute and relative frequencies. Survival times were compared between groups using the Kaplan-Meier curves and the Mantel-Cox log-rank test. The Cox regression analysis was used to adjust survival for continuous variables. Dichotomous outcomes were adjusted using binary logistic regression. Simple associations between categorical variables were assessed using Pearson’s chi-square test. Continuous variables were compared between groups using the Kruskal-Wallis test. Survival time was defined as the interval of time from the date of surgery to the date of death. Statistical significance level of 95% was p<0.05. The research protocol was approved by the ethics committee of the São João University Hospital, approval number CE-03-22, and was exempted from obtaining patients’ consent due to its retrospective nature. Access to clinical data was authorized by the Responsible for Access to Information.

## RESULTS

A total of 134 patients with locally advanced GC were evaluated. The characteristics of the patients, tumor, POC, and surgical resection are shown in Table 1. The median age was 64 (55–70) years and 56.0% of the patients were male. Regarding the location of the tumors, 48.5% were located in the antrum, 40.3% in the body of the stomach, and 4.5% in the cardia (87.3% of the gastric tumors were adenocarcinomas). The most frequently used regimes of POC were FLOT (36.6%), EOX (35.1%), and MDCF (14.9%). The median number of POC cycles was 3 (3–4). The percentage of R0 resection was 89.6%.

**Table 1.** Characteristics of patients, tumor, perioperative chemotherapy, and surgical resection.

	Variable	p-value (%)
Age (years)	Median (range)	64 (55–70)
Gender	Masculine	75 (56.0)
	Feminine	59 (44.0)
Tumor location	Antrum	65 (48.5)
	Body	54 (40.3)
	Fundus	2 (1.5)
	Diffuse	4 (3.0)
	Gastric remnant	2 (1.5)
	Cardia	6 (45)
Histological type	Adenocarcinoma	117 (87.3)
	Others	17 (12.7)
Perioperative chemotherapy regimen	ECF	2 (1.5)
	EOX	47 (35.1)
	CF	2 (1.5)
	MDCF	20 (14.9)
	FOLFOX	10 (7.5)
	TPF	2 (1.5)
	XP	1 (0.7)
	XELOX	1 (0.7)
	FLOT	49 (36.6)
	Total	134
Number of preoperative chemotherapy cycles	Median (range)	3 (3–4)
Surgical resection	R0	120 (89.6)
	R1	13 (9.7)
	R2	1 (0.7)
	Total	134

### Pathological response of the tumor at the primary site

The histological response of the primary tumor after POC is shown in Table 2. It is observed that the most common histological stage in our sample is IIA (17.9%), followed by IV (14.9%), IIB (13.4%), and IIIA (12.7%). The omitted case corresponds to an yTxN3bR0 patient.

**Table 2.** Anatomopathological characteristics, histological staging, and operative morbidity.

	Variable	p-value (%)
Number of resected lymph nodes	Median (IQR)	27 (19.0–36.3)
Number of invaded lymph nodes	Median (IQR)	2 (0–9.5)
Number of regressed lymph nodes	Median (IQR)	1 (0–4)
Number of patients with invaded lymph nodes	No	48 (35.8)
	Yes	86 (64.2)
	Total	134
Number of patients with regressed lymph nodes	No	48 (35.8)
	Yes	62 (46.3)
	Total	110
	Omitted	24
Proportion of regressed lymph nodes		41.9%
Becker’s criteria	TRG 1a – complete tumor regression	3 (2.2)
	TRG 1b – <10 % residual tumor	27 (20.1)
	TRG 2 – 10–50 % residual tumor	33 (24.6)
	TRG 3 – >50% residual tumor	66 (49.3)
	Total	129 (96.3)
	Omitted	5
Histological staging	Stage 0	2 (1.5)
	Stage IA	10 (7.5)
	Stage IB	13 (9.7)
	Stage IIA	24 (17.9)
	Stage IIB	18 (13.4)
	Stage IIIA	17 (12.7)
	Stage IIIB	15 (11.2)
	Stage IIIC	14 (10.4)
	Stage IV	20 (14.9)
	Total	134 (99.3)
	Omitted	1
Lymphatic invasion	No	59 (44.4)
	Yes	74 (55.2)
	Total	133 (99.3)
	Omitted	1
Venous invasion	No	83 (61.9)
	Yes	49 (36.6)
	Total	132 (98.5)
	Omitted	2
Operative morbidity	No	93 (69.4)
	Yes	41 (30.6)
	Total	134

IQR: interquartile range; TGR: tumor regression grading.

The analysis between the different grades of Becker’s criteria and the histological stage (Table 3) showed that: of the 3 patients with TRG 1a, 2 (66.7%) had stage 0, while 1 (33.3%) had stage IV; of the 26 patients with TRG 1b, the most common histological stage was IIA, present in 12 (46.2%) patients; of the 33 patients with TRG 2, 10 (30.3%) patients presented with stage IIIA, while 4 (12.1%) had stage IIIC. There was a correlation between the variables of Becker’s criteria and histological stage (p<0.001). The same is true for Becker’s criteria and lymphatic and venous invasion (p<0.001 and 0.002, respectively).

**Table 3.** Cross-tabulation: Becker’s criteria, histological staging, lymphatic, and venous invasion.

Becker’s criteria		TRG 1a	TRG 1b	TRG 2	TRG 3	p-value
Histological staging	n (%) stage 0	2 (66.7%)	0 (0%)	0 (0%)	0 (0%)	<0.001
	n (%) stage IA	0 (0%)	3 (11.5%)	2 (6.1%)	5 (7.6%)	
	n (%) stage IB	0 (0%)	3 (11.5%)	1 (3.0%)	8 (12.1%)	
	n (%) stage IIA	0 (0%)	12 (46.2%)	2 (6.1%)	10 (15.2%)	
	n (%) stage IIB	0 (0%)	1 (3.8%)	5 (15.2%)	10 (15.2%)	
	n (%) stage IIIA	0 (0%)	2 (7.7%)	10 (30.3%)	5 (7.6%)	
	n (%) stage IIIB	0 (0%)	2 (7.7%)	4 (12.1%)	8 (12.1%)	
	n (%) stage IIIC	0 (0%)	0 (0%)	2 (6.1%)	11 (16.7%)	
	n (%) stage IV	1 (33.3%)	3 (11.5%)	7 (21.2%)	9 (13.6%)	
Lymphatic invasion	No	2 (100%)	22 (81.5%)	10 (30.3%)	24 (36.4%)	<0.001
	Yes	0 (0%)	5 (18.5%)	23 (69.7%)	42 (63.6%)	
Venous invasion	No	2 (100%)	25 (92.6%)	18 (54.5%)	35 (53.8%)	0.002
	Yes	0 (0%)	2 (7.4%)	15 (45.5%)	30 (46.2%)	

p-values were determined through the Fisher’s exact test. N: number of patients; TRG 1a: complete tumor regression (absence of tumor); TRG 1b: subtotal tumor regression (<10% of residual tumor); TRG 2: partial tumor regression (10–50% of residual tumor); TRG 3: minimal or absent tumor regression (>50% of residual tumor).

### Pathological response of lymph nodes

In all, 86 (64.2%) patients had LN invasion. In 110 patients, 62 (46.3%) showed signs of LN regression. The proportion of regressed LNs was 41.9%. In 133 patients, 74 (55.2%) presented with lymphatic invasion, and in 132 patients, 49 (36.6%) showed venous invasion.

### Postoperative morbidity

Postoperative complications occurred in 41 (30.6%) cases. We can conclude that the morbidity regarding the number of resected LNs has a value of 1.001, which means that the odds of developing postoperative complications increased by 1.001 (0.974–1.029) for each resected node. Regarding regressed LNs, the odds of developing complications increase to 1.043 (0.960–1.133) for each resected node. In the case of Becker’s criteria, these odds increase by 1.247 (0.791–1.996) for each stage. In more serious complications (Clavien-Dindo ^3^IIIa), the odds rise by 0.007 (0.271–1.673) for each Becker stage.

The cases were divided into two groups, in which TRG 1a and TRG 1b were defined as good responders, and TRG 2 and TRG 3 as poor responders to preoperative therapy.

As can be seen in Table 4, the variables of good and poor responders and the surgical complications were independent of each other. The same was seen regarding good and bad responders and morbidity (≥3 in the Clavien-Dindo classification).

**Table 4.** Cross-tabulation: responders, postoperative complications, and Clavien-Dindo classification.

Responders	Postoperative complications		Clavien-Dindo ≥IIIa	
	No	Yes	No	Yes
Good responders	67 (67.7%)	32 (32.3%)	17 (60.7%)	11 (39.3%)
Poor responders	24 (80.0%)	6 (20.0%)	3 (50.0%)	3 (50.0%)
p-value	1.683		0.234	

p-values were determined through the Fisher’s exact test.

**Figure 1.**
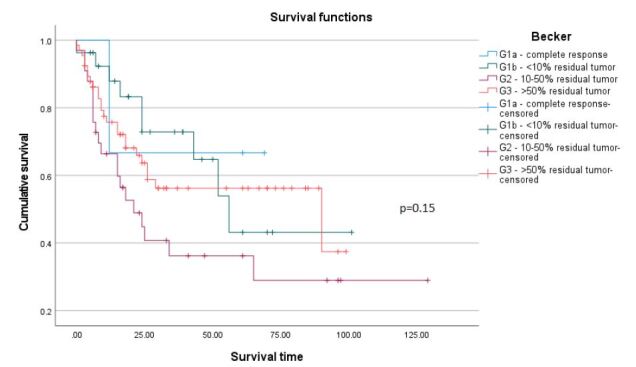

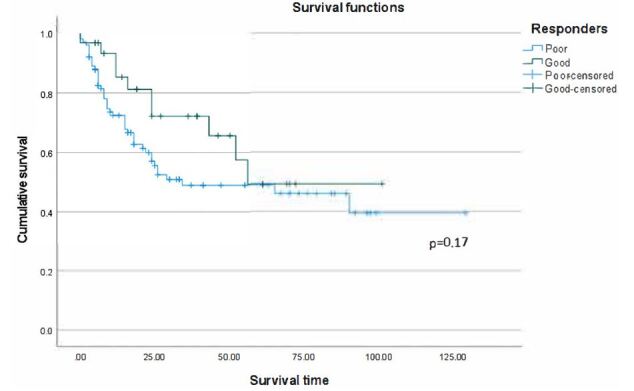
Cumulative survival rate according to tumor regression grading (TRG) (A) and good and poor responders (B). Differences in cumulative survival rates were determined through the Kaplan-Meier method and compared using the log-rank test. The level of significance considered was p<0.05.

The median time of follow-up was 56.0 (13.2–98.8) months. Figure 1A shows that the 5-year survival rate is higher in patients in the TRG 3 group when compared to those in the TRG 1b and TRG 2 groups. However, these last two have a reduced number of cases, which might alter the results. Figure 1B shows that the good responders group has a median survival of 56.0 months, while the group of poor responders has a median survival of 34.0 months, with p=0.17.

A – Tumor regression grade

B – Poor and good responders

## DISCUSSION

GC is an aggressive neoplasm with a poor prognosis, mostly due to the absence of symptoms in early stages, often resulting in late diagnosis. In the past decades, the multimodal approach with POC has been widely determined as standard treatment in most European countries^18^, since it increases the survival of patients with advanced GC. However, despite this approach, it is estimated that R0 resection is only achieved in 40–50% of the cases^28^, meaning that most patients will have a relapse or die due to this neoplasm^16^. This can be explained by the aggressive biological behavior of this neoplasm, characteristics of the patients (such as an advanced stage at the moment of diagnosis, advanced age, high BMI, the presence of multiple comorbidities)^8^, and the presence of occult micrometastases, which increases the probability of early invasion of LN^1^. Although the surgical approach in early stages of GC (T1N0) shows favorable results, survival is drastically reduced when neoplastic cells infiltrate past the submucosa or when there is LN invasion^24^.

TRG is one of the morphological parameters that can be used to assess the effect of POC on the primary tumor. There are at least five rating systems for evaluating the TRG: TRG-Mandard, TRG-JGCA, TRG-CAP, TRG-Becker, and TRG-China. In this study, the classification system used was the one proposed by Becker et al. in 2003^5^, and it validated and determined as an independent prognostic factor for locally advanced GC in 2011^8,10,29^.

According to our analysis, only 22.3% of the patients who underwent POC had TRG 1a or TRG 1b, meaning that they were classified as good responders, whereas 75.9% were considered poor responders. These values are similar to those reported in previous studies, such as those by Smyth et al.^16^, Lombardi et al.^8^, Becker et al.^5^, and Schmidt et al.^14^.

We found that 64.2% of our patients had invaded LN, while 46.3% had regressed LN. On the contrary, 49.4% had no lymphatic invasion and 61.9% had no signs of venous invasion after POC. These histopathological findings are in agreement with those reported in the literature^6,11,25^. Charruf et al.^6^ demonstrated that the group of patients subjected to neoadjuvant chemotherapy (NAC) had smaller tumor sizes, less lymphatic, venous and perineural invasion, and a higher percentage of patients with pT1 and T2 and lower pN0, when compared to the group subjected only to surgery. It is important to point out that, in the group subjected to NAC, 60 and 82.2% of the patients had no lymphatic and venous invasion, respectively. The persistence of metastases in LN is an indicator of poor prognosis. Thus, ypN0 patients with or without LN metastases before POC had a similar prognosis^11,30^. Pereira et al.^12^ demonstrated that the staging of LNs is a more important prognostic factor than the regression of the primary tumor since they found that patients with a higher rate of LN regression had greater survival than those with a low rate of LN regression. On the contrary, patients considered good responders did not have a statistically significant higher survival when compared to the poor responders. Furthermore, this study also showed that patients with high rates of LN regression had primary tumors with smaller depths and diameters and the absence of venous, lymphatic, or perineural invasion.

Despite the benefits of POC, there are controversial data regarding the possible increase in perioperative morbidity and mortality, due to its toxicity and the consequent worsening of the patient’s nutritional profile (namely sarcopenia)^26^. Luo et al.^9^ pointed out several possible explanations for the contradictory data present in the literature: most studies report morbidity and mortality as secondary end points in their studies; there are different NAC regimens applied, which hinder the assessment of postoperative complications specific to a given NAC regimen; and there are variations in both the definition and classification used for postoperative complications. In this study, postoperative morbidity was present in 30.6% of the patients. Furthermore, we concluded that the variables “good and poor responders” and “surgical complications” were independent of each other.

Ahn et al.^2^ compared two groups of patients, one subjected to NAC and the other to surgery alone. They concluded that there were no statistically significant differences in the morbidity, mortality, and reoperation rates between the two groups. The rate of R0 surgical resection in the group who underwent NAC was 92.2%. This value is very similar to that in our study. Wu et al.^26^ also showed that there were no statistically significant differences in postoperative morbidity and mortality, regardless of type or severity. In their study, in the group subjected to NAC and surgery, 28.7% of the patients had operative morbidity and 9.2% had morbidity greater or equal to IIIa according to the Clavien-Dindo classification. Yan et al.^27^ also did not show statistically significant rates of postoperative morbidity, since the value found was 29.9%. These sets of results are confirmed by our series of patients, although they are opposite to the ones presented in the CRITICS trial. Umeda et al.^23^ also had similar results to the aforementioned studies, where the rates of complications superior to IIIa (Clavien-Dindo classification), reoperation, and rehospitalization between the groups submitted to NAC versus non-NAC showed no statistically significant differences. However, in the group that underwent NAC, blood loss and surgery time were significantly higher.

Charruf et al.^6^ demonstrated that major postoperative complications (superior to IIIa in the Clavien-Dindo classification) were significantly less in the groups submitted to NAC versus the ones who underwent surgery alone. According to the authors, a possible explanation is the reduction in the volume of the tumor brought about by NAC, which allows for less extensive surgical procedures/approaches when compared to the group submitted to surgery alone.

By analyzing survival and TRG, we found that the group of patients with TRG2 showed lower survival rates. On the one hand, these results can be explained by the fact that 23 (69.7%) and 15 (45.5%) of the patients showed lymphatic and venous invasion, respectively. Consequently, and according to the literature, these lead to a higher risk of recurrence and a lower prognosis^6,11,25^. On the other hand, most of the patients in this group had a histological stage equal to IIIA or IV (Table 3).

According to Charruf et al.^6^, the TRG was not statistically related to patient survival – the authors hypothesize that the primary tumor’s biology might be different from that of LN. In their study, patients with pN0 who underwent NAC or surgery had the same survival, highlighting the importance of the absence of LN metastases in survival. In this article, the authors do not show the TRG, but they present the values of cN+ and pN+. In our study, using the aforementioned formula, we determined that the proportion of regressed LNs was 41.9%.

Woodham et al.^25^ showed that lymphatic, perineural, and venous invasions after NAC were related to reduced survival and that the presence of one form of invasion increased the risk of the other forms being present, meaning that survival decreased as these histopathological factors increase.

The results of our study should be analyzed taking into account its limitations, namely: it is a retrospective study; our sample is small (134 cases); different regimens of POC are used, which can hinder the interpretation of results; the long interval of time in which the study takes place, during which changes may have occurred both in the treatment of patients and in clinical staging; and the fact that the study was carried out in a single institution, meaning that extrapolations from our results should be done with caution.

## CONCLUSIONS

Our study confirms that POC induces the regression of both the primary tumor and LNs. The findings regarding operative morbidity were similar to those described in the literature. Nevertheless, although the group of good responders showed higher survival, the difference was not statistically significant when compared to the group of poor responders. Thus, there is a need for further studies that assess the importance of TRG and its impact on the survival of these patients.
